# Evaluation and Defect Detection in L-Shaped GFRP Laminates by Infrared Thermography

**DOI:** 10.3390/ma17122830

**Published:** 2024-06-10

**Authors:** Małgorzata Chwał, Adam Stawiarski, Marek Barski, Marcin Augustyn

**Affiliations:** Department of Machine Design and Composite Structures, Faculty of Mechanical Engineering, Cracow University of Technology, Al. Jana Pawła II 37, 31-864 Kraków, Poland; adam.stawiarski@pk.edu.pl (A.S.); marek.barski@pk.edu.pl (M.B.); marcin.augustyn@pk.edu.pl (M.A.)

**Keywords:** GFRP laminates, defects, wrinkles, thermography, product quality, manufacturing

## Abstract

Glass fiber-reinforced polymer (GFRP) laminates are used in many applications because of their availability, high mechanical properties, and cost-effectiveness. Fiber defects in the form of waviness or wrinkles can occur during the production of multilayered laminates. When curved laminates of significant thickness are produced, the likelihood of such defects increases. Studies have confirmed that fiber deformation during manufacture leads to a reduction in the mechanical properties of laminates. Therefore, early detection of such defects is essential. The main part of this paper deals with research into the possibility of using active infrared thermography to detect wrinkles in curved multilayered GFRP laminates. The size of the artificial wrinkles was assessed by analyzing scans and microimages. The shape deformations of the samples were evaluated by comparing the samples with the mold and the assumed nominal shape. The influence of the out-of-autoclave manufacturing process on the reduction in wrinkles formed without significantly affecting the internal structure of the laminate is presented in this work. This research demonstrated the ability to detect wrinkles in thick curved laminates using active infrared thermography. However, it also showed how the interpretation of the thermographic results is affected by the curvature of the structure, the lack of uniform heating, and the configuration of the thermographic setup.

## 1. Introduction

Laminate composites are widely used in aerospace engineering, shipbuilding, the tank industry, the automotive industry, etc. Fiber-filled composites offer properties such as high specific strength and stiffness, high corrosion resistance, and low mass, which are much higher than the properties of classical materials. Curved laminates are commonly used in elements of wind turbine systems or aerospace structures [1,2]. The variety of fiber materials, orientations, configurations, and manufacturing methods used in multilayered composites makes the material highly susceptible to defects that occur during manufacturing, processing, and use. When evaluating the quality of a product, the most important thing to consider is the manufacturing defects that have a very high probability of occurring. Therefore, the study of manufacturing defects and the search for detection methods useful for polymeric laminates are currently under investigation [3,4,5,6,7]. A comprehensive review of manufacturing defects and their detection methods was presented by Fu and Yao [8] and Azzouz et al. [9].

During the manufacture of fiber-reinforced composites, defects such as fiber wrinkles, waviness and breakage, air voids, other material inclusions, resin contact areas, and delamination can occur [10,11,12,13,14,15]. Compared with flat structures, composites with curved shapes are subjected to much higher bending and torsional loads in service. The curved L- and C-shaped laminates tend to change their properties locally in the corner area [16]. The corner is the location where thickness variation is localized because of the shape of the mold, corner radius, material type, stacking sequence, and assumed number of plies [11].

Two types of failure mechanisms are more likely to occur in curved multilayer structures. Intralaminar cracking initiates and propagates in the matrix. This cracking leads to interlaminar cracking between the layers (delamination) [17]. For curved laminates, interlaminar failure is dominant, while intralaminar failure is only observed in cross-play multilayered composites [18,19].

Previous analyses of the mechanical performance of laminates with manufacturing defects revealed a reduction in the modulus, strength, and reliability of their structures [20,21,22,23,24]. These studies were carried out under static [18,19] and fatigue loading [18,25,26]. The experimental and numerical analysis of curved laminates has been carried out in many papers, e.g., [27,28,29,30,31]. Woo et al. [32] experimentally and numerically analyzed the influence of manufacturing defects on the delamination behavior of carbon fiber (CFRP) L-shaped composite beams. They considered the effect of wavy plies, the presence of pure resin, and the stacking sequence. Cinar [23] analyzed the influence of fiber waviness on the strength of glass fiber (GFRP) laminates through bending tests. 

An analysis of the literature in the area of curved laminates shows that most of the work is focused on carbon fiber-based composites in autoclave techniques, e.g., [16,32]. Out-of-autoclave manufacturing techniques have recently attracted increasing interest in the fabrication of more complex and larger multilayered composite parts [11,15,23]. Wet hand lay-up is still a technique that offers high consistency and lower labor costs compared with prepreg application or infusion techniques. Hand lay-up supported by the vacuum bagging method produces high-quality products at relatively low production costs.

In general, multilayered composites can be constructed either from unidirectional plies or by stacking different types of textile composites [23,33]. Most papers on curved laminates consider prepreg systems with different configurations. For example, the vacuum-bagged L-shaped laminate consisting of braided carbon/epoxy composite plies has been considered in papers [4,15,33]. Textile composites are prone to wrinkling because of the high coefficient of friction between tows and inter-ply interactions [34], whereas fiber wrinkling in UD prepreg material is mostly caused by slippage between plies [35]. In laminates with complex geometries, it is not possible to eliminate defects such as fiber waviness and wrinkles. Therefore, their influence on the mechanical behavior of multilayered structures should be taken into account. In addition, detection methods for manufacturing defects should be developed.

Only a few papers in the field of wrinkles in curved laminates deal with glass fiber-based composites, e.g., the influence of fiber waviness on the strength of L-shaped glass laminates was analyzed in the paper [23]. However, glass fibers are used in many applications. They are used to make airplane and glider fuselages, car body panels, and ship hulls. In the chemical industry, they are used to build high-pressure tanks and pipes. Composite materials reinforced with these fibers can also be found in electrical and construction equipment as well as sports and recreational equipment. Glass laminates are used in many curved wind turbine blade components. The same is true for components with complex shapes, such as those used in the marine industry.

Manufacturing defects, which have a very high probability of occurrence, are the most important in assessing laminate quality. Therefore, the search for detection methods useful for polymeric laminates is still the main focus of many scientific papers. To date, there are only a few non-destructive methods for defect detection in multilayer structures [36], i.e., acoustic emission, ultrasonic inspection, eddy current testing, infrared thermography, shearography, computed tomography, and digital image correlation. The actual progress in the field of non-destructive testing and evaluation techniques of composite defects is described in the paper by Chen et al. [37]. For the detection of defects in the form of fiber waviness and wrinkles in laminates, ultrasonic inspection [5,38], eddy current method [39], and infrared thermography [7] are used. 

Infrared thermography has great potential for laminate defect detection because of its high inspection speed, real-time and full-field defect visualization, safe and easy operation, and cost-effectiveness. However, the sensitivity is lower for defects deeper below the surface, and in active thermography, there is a possible risk of thermal destruction of the sample being inspected. To date, infrared thermography has been successfully applied in the detection and evaluation of voids, delamination, debonding, fiber-matrix cracking, and impact damage [37]. The application of this method to the detection of defects in the form of waviness and wrinkling of fibers in laminates is still under development [7]. Therefore, the analysis of artificial wrinkle defects can help to develop a qualitative and quantitative evaluation of IRT techniques and find their limitations. Current trends in NDT show the potential of using combined techniques, such as infrared thermography and ultrasonic testing, to increase the accuracy of detection [37]. 

The current study is concerned with an artificial wrinkle created in the corner of a curved sample of a glass–epoxy L-shaped laminate composite. The main part of this work focuses on the possibility of detecting wrinkles in curved laminates using active infrared thermography. An analysis of microimages of the sample and a 3D scan was used to characterize the wrinkles in the case of hand lay-up and vacuum bagging techniques. After a literature review, the topic was selected based on the lack of studies on manufacturing defects in the form of wrinkles in curved fiberglass laminates manufactured using out-of-autoclave techniques. 

## 2. Materials and Methods

**Figure 1:**
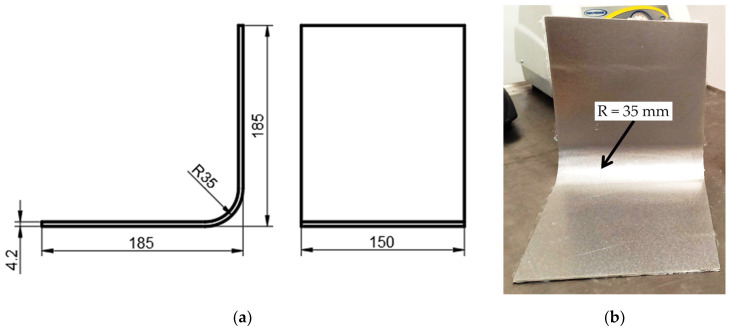
L-shaped laminate: (**a**) nominal dimensions of a laminated sample and (**b**) the mold applied in the manufacturing.

The analysis focuses on L-shaped laminates made of glass fiber-reinforced epoxy resin. The samples consist of 16 layers of glass twill fabric produced by the hand lay-up and vacuum bagging techniques. The assumed nominal thickness of the samples is 4.2 mm. A mold was made from a steel sheet bent into an L-shaped part with an inner radius equal to R = 35 mm. Figure 1 shows the nominal dimensions of a laminated sample and the mold used in its manufacture.

**Figure 2:**
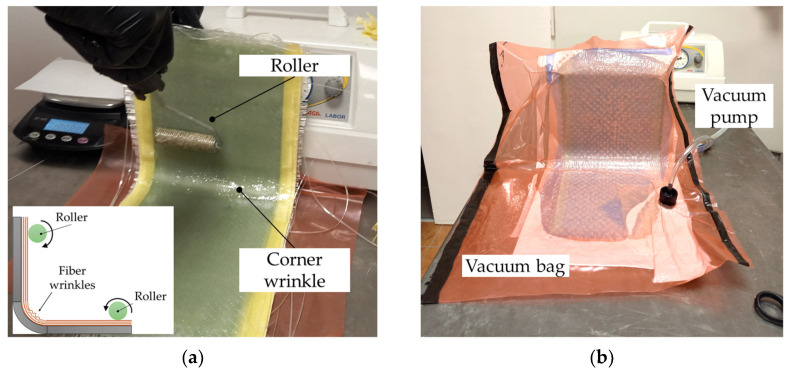
Manufacturing of L-shaped laminates: (**a**) formation of an artificial wrinkle and (**b**) vacuum bagging.

The fiberglass layers were impregnated with resin on a flat plate during the hand lay-up process. Next, the entire stack of wet layers was bent on the concave side of the mold, and a wrinkle was formed in the corner while pressing the layers with a roller—Figure 2a. The sample was allowed to cure at room temperature. The first stage of the vacuum bagging technique was similar to the open mold hand lay-up. The mold with wet plies was then placed in a vacuum bag with additional materials such as release fabric, perforated film, and breather material, and connected to a vacuum pump to consolidate the laminate and pump out excess epoxy—Figure 2b. A vacuum of approximately −0.9 bar was applied.

**Figure 3:**
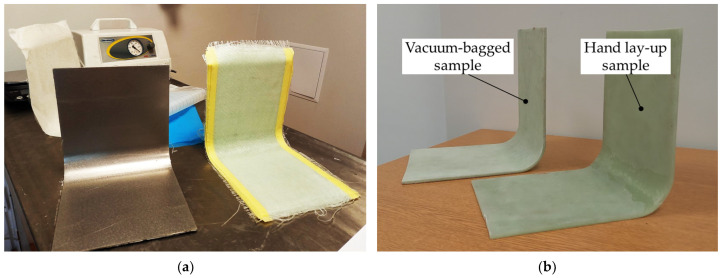
L-shaped samples: (**a**) the mold and hand lay-up samples before cutting and (**b**) the hand lay-up sample and vacuum-bagged samples.

A vacuum can help remove air voids and increase the fiber-to-epoxy ratio in the laminate; therefore, the average measured thickness of the vacuum-bagged sample of the straight arm was 4.2 mm, while the thickness of the hand lay-up sample was approximately 4.9 mm. By controlling the weight of fiber and resin during the fabrication process, the fiber volume fraction was estimated to be 58% for the vacuum-bagged sample and 43% for the hand lay-up sample. The L-shaped samples are shown in Figure 3. A wrinkle is visible in the hand lay-up sample, while the wrinkle formed in the vacuum-bagged sample is almost invisible. The vacuum caused the artificially formed wrinkles to flatten out.

**Figure 4:**
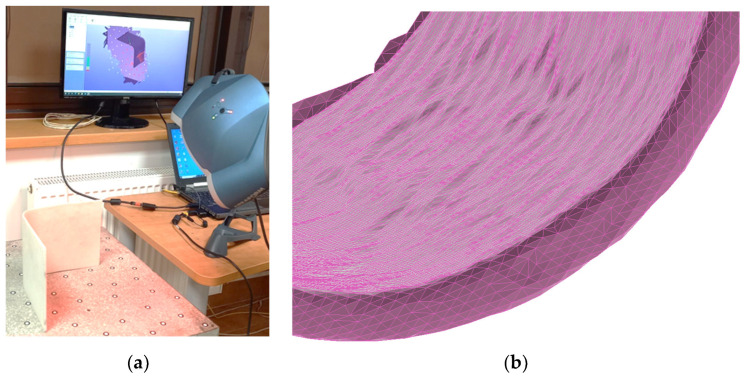
Quality inspection of L-shaped samples: (**a**) 3D scan of the object and (**b**) triangular facet mapping of the laminate shape.

To assess the quality of the samples, L-shaped composites were scanned. The 3D Creaform REVscan laser scanner (Amtek Company, Arnold, MD, USA) was used to digitize the sample. The point cloud was converted to the triangle mesh with the assumed accuracy. GOM Inspect 2022 SP1 software was used to validate the quality of the manufactured digitized parts with respect to the nominal CAD model. The Creaform REVscan is a self-positioning, handheld scanner used for inspection, quality control, and reverse engineering measurements. The scanner is capable of digitizing 18,000 points per second with an accuracy of up to 50 µm. The 3D scanning of the sample and the triangular facet mapping of the laminate shape are shown in Figure 4a and Figure 4b, respectively.

In the present work, a non-destructive technique such as active infrared thermography (IRT) is used to investigate the possibility of detecting wrinkles in curved GFRP composite samples. The work by Stawiarski et al. [7], which focused on the detection of wrinkles in plates and in the turbine blade, demonstrated the effectiveness of IRT in such analyses. Active thermography is a real-time, full-field detection method that generally involves the generation and propagation of heat flow into the test object and the detection of thermal responses. In this method, the external heat source generates an internal heat flow and temperature rise to create a relevant thermal contrast between areas of interest.

**Figure 5:**
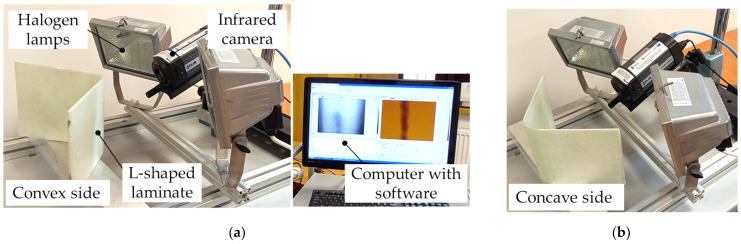
Experimental setup for the thermographic analysis of L-shaped samples: (**a**) convex side and (**b**) concave side.

The reflection position of the halogen lamps was used to generate the heat flux. The IR camera and halogen lamps are installed on the same side in the reflection technique. The IRT setup was installed in the reflection mode to approximate real-operated monitoring system conditions where an operator cannot place an analyzed structure between the heat source and the IR camera—Figure 5. If there is easy access to both sides of the object, the transmission technique is also used in thermographic analysis [7].

The thermographic analyses were conducted in two variants of the L-shaped structure configurations in the experimental setup, i.e., the sample was heated from its convex and concave sides—Figure 5a,b. From the convex side of the L-shaped sample, the artificial wrinkle is invisible to the operator.

In this study, the sample was heated with the use of two halogen lamps at a distance from the sample—Figure 5. Lamps of a preset configuration were operated at full heating power for a specified period. During both the heating and cooling of the sample, the IR camera recorded the temperature distribution on the surface of the sample. The data collected by the IR camera were then processed and presented in the form of thermal curves.

The infrared camera used was the FLIR A325 (FLIR Systems, Wilsonville, OR, USA) with the following characteristics: resolution 320 × 240, frame rate 60 Hz, spectral range 7.5–13 μm, and temperature range −20–+350 °C. The rectangular thermal signal from two 500 W halogen lamps was used to generate thermal contrast in the samples. The entire IRT test was set to 120 s, while the heating lasted 30 s with a frame rate of 9 Hz. This means that the thermal response of the analyzed structures was monitored during both the heating and cooling processes. The data were processed using IrNDT v1.7.2 software and ThermaCAM Researcher Pro 2.10 software. Transient thermal analysis with the same analysis conditions was applied to both samples.

## 3. Results

The quality of samples and the possibility of wrinkle defect detection of the L-shaped composite structures were analyzed based on the 3D scans, microscopic observations, and thermal image processing.

### 3.1. Part Quality

**Figure 6:**
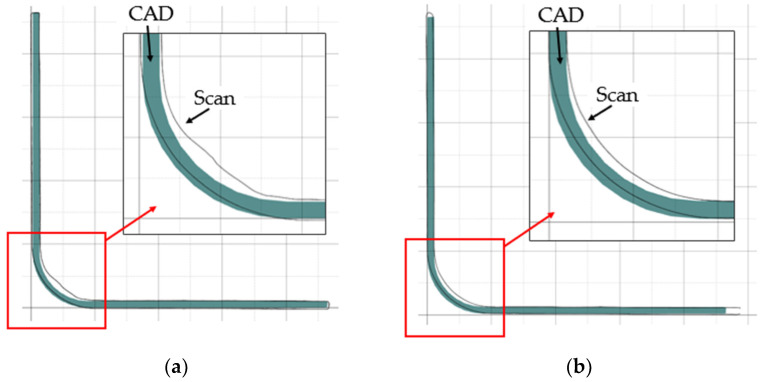

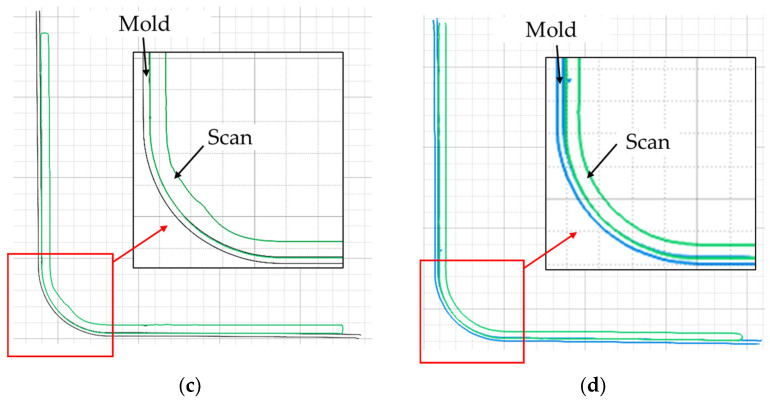
Cross-section comparisons: (**a**) nominal CAD model and 3D scan (gray lines) for the hand lay-up sample; (**b**) nominal CAD model and 3D scan (gray lines) for the vacuum-bagged sample; (**c**) mold to 3D scan (green lines) for the hand lay-up sample; and (**d**) mold to 3D scan (green lines) for the vacuum-bagged sample.

**Figure 7:**
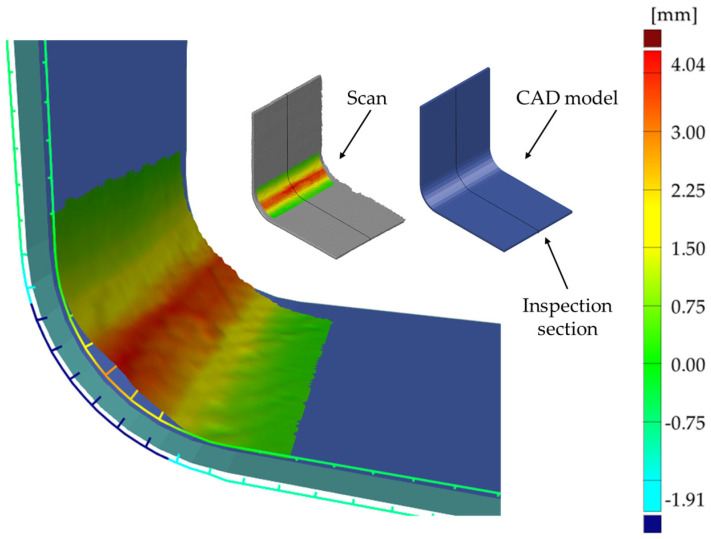
Comparison of the 3D scan to the nominal CAD model for the hand lay-up sample.

**Figure 8:**
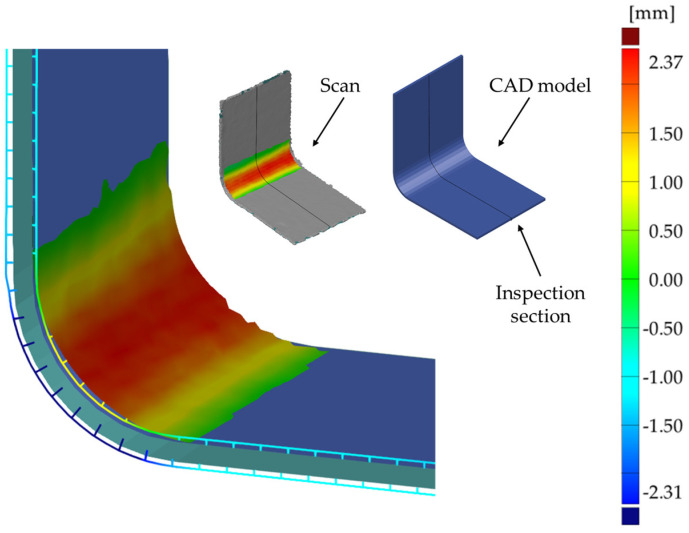
Comparison of the 3D scan to the nominal CAD model for the vacuum-bagged sample.

A comparison of the nominal CAD model (dimensions in Figure 1a) with the 3D scan for both the hand lay-up and vacuum-bagged samples is shown in Figure 6, Figure 7 and Figure 8. 

The assumed nominal thickness of the CAD model of the 16-layer laminate is 4.2 mm, while the average thickness of the real samples measured on the straight arms was 4.9 mm and 4.2 mm for the hand lay-up and vacuum-bagged sample, respectively. 

Comparison of the nominal CAD model with the actual 3D scan revealed not only wrinkles but also shape deformation of the L-shaped laminate. An example comparison of the inspection cross-section of the CAD model and the scan is shown in Figure 6. For both samples, the largest shape distortion is seen on the convex side at the contact area with the mold. There is less deformation in this area for the hand lay-up sample—Figure 6a. 

To verify the shape deviations in the samples relative to the shape of the mold, the mold scan was compared to the sample scan. The results are shown in Figure 6c,d. As one might expect, the comparison of these scans shows a very good match between the sample and the mold. Small deviations are only visible at the ends of the flat areas of the samples.

The comparison of the sample scans with the nominal CAD model presented in Figure 6a,b shows significant sample shape deviations in the curved area from the assumed ideal shape of the part. These shape deviations were influenced by both mold design errors and the presence of wrinkles.

In general, manufactured products should relate to the assumed nominal shape of the part, so Figure 7 and Figure 8 show the scan comparison to the nominal CAD model of the part. With a near-perfect match between the convex surface of the samples and the mold, it was deemed useful to show how the scan deviated from the assumed ideal shape of the samples.

The deviation in the scanned shape to the nominal CAD model is less than 2 mm—Figure 7. The shape differences decrease in the flat arms of the sample. For the vacuum-bagged sample, the deviation in the corner of the sample from the mold contact side is higher than for the hand lay-up sample and is more than 2 mm—Figure 6b and Figure 8.

A thicker, wider, and more corrugated wrinkle is visible for the concave side of the hand lay-up sample—Figure 3b and Figure 7. The artificial wrinkle locally increases the thickness of the sample by approximately 2 mm at the corner. In the vacuum bagging technique, the identically created artificial wrinkle was significantly reduced in size under the pressure of the vacuum. After the manufacturing process, the wrinkle was almost completely flattened—Figure 3b and Figure 8. This indicates that the parameters of the manufacturing process have a strong influence on the possibility of spontaneous wrinkle elimination that occurs unintentionally when wet layers are applied to the mold. 

**Figure 9:**
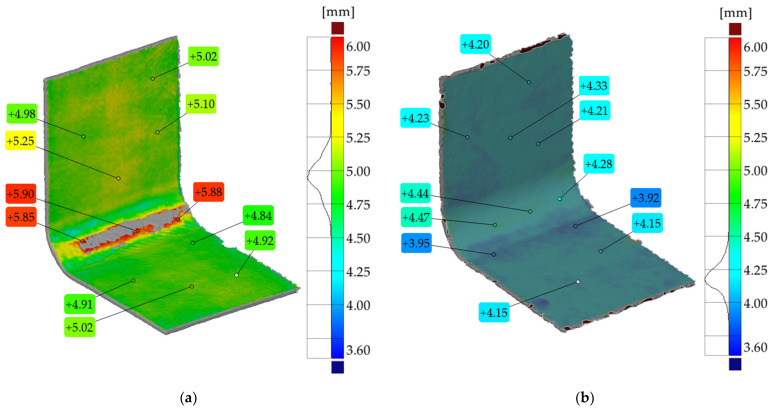
Thickness distribution: (**a**) hand lay-up sample and (**b**) vacuum-bagged sample.

The thickness distribution of the samples is shown in Figure 9. The sample manufactured by the hand lay-up technique has an extensive wrinkle in its corner. The average thickness of the wrinkle is more than 2 mm, while the average thickness of the sample is 4.9 mm—Figure 9a. Figure 9a shows the limited thickness of the sample in the wrinkle area to better visualize the non-uniform thickness distribution for the entire sample. 

The full appearance of the wrinkle is shown in Figure 7, and its outline is shown in Figure 6a. The sample produced by the vacuum bagging technique has a more uniform thickness distribution. The artificially introduced wrinkle was flattened by the vacuum. A small footprint of the presence of the wrinkle in the corner of the sample is manifested by a local increase in the thickness of about 0.3 mm—Figure 9b. The average thickness of the sample is as expected and is 4.2 mm.

### 3.2. Microscopic Observations

**Figure 10:**
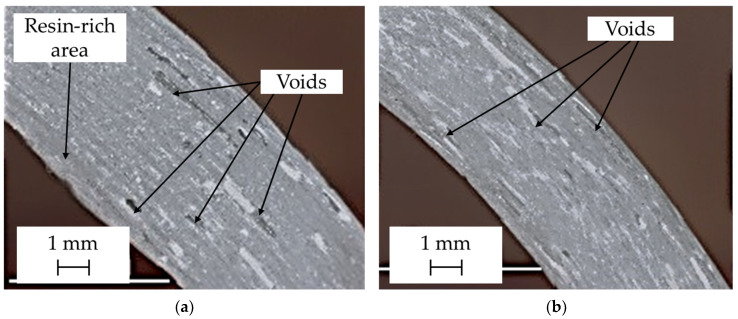
Microimages of the curved area: (**a**) hand lay-up sample and (**b**) vacuum-bagged sample.

The morphology of the L-shaped samples was revealed by a series of images taken with a CMOS digital microscope (Delta Optical, Warsaw, Poland). The most important is the corner of the curved sample where the artificial wrinkle was created. The selected images shown in Figure 10 were taken from the front surface of the curved part of the sample. Both microimages clearly show defects in the form of voids. The resin-rich area and larger voids are particularly visible in the curved area of the hand lay-up sample—Figure 10a. Excess resin and large voids were created by pressing the wet layers with a roller and moving the roller toward the corner. Smaller voids are visible in the vacuum-bagged sample—Figure 10b.

### 3.3. Infrared Thermography

The main objective of this paper is to determine the effectiveness of IR thermography in detecting wrinkles in curved laminates. Since the quality of the product depends on the method used and the parameters of the manufacturing process (as shown above—Figure 10), active infrared thermography was applied to both the hand lay-up and vacuum bagging methods.

**Figure 11:**
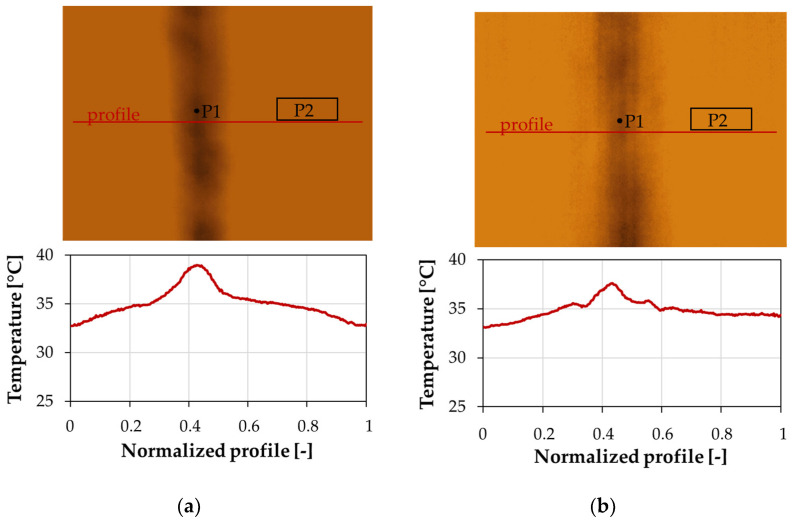
The thermal images and temperature distribution along the profile for the hand lay-up sample at the end of the heating time: (**a**) convex side and (**b**) concave side.

**Figure 12:**
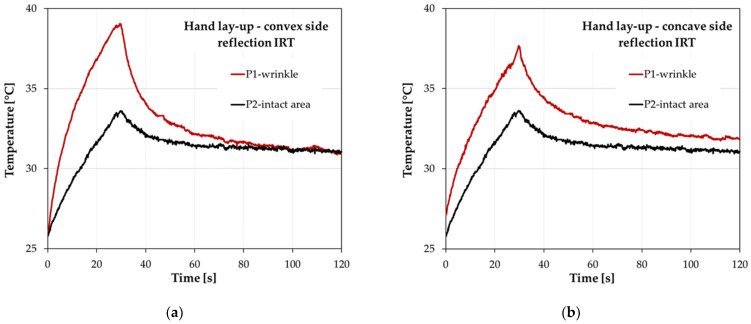
Temperature distribution during analysis for the hand lay-up sample for the wrinkle (point P1) and the reference area (point P2): (**a**) convex side and (**b**) concave side.

The thermal images and thermographic results in the form of temperature changes during the heating and cooling of the sample for the areas of interest are presented in graphical form. The reflection IRT results for the hand lay-up sample are shown in Figure 11 and Figure 12. Figure 11 shows the thermal images and temperature distribution at 30 s of analysis (end of the heating—beginning of the natural cooling) along the normalized measurement profile for a hand lay-up sample in the case of the convex and concave orientation of the sample with respect to the IR camera shown in Figure 5. Wrinkles were detected for both sample orientations. 

In the thermal images, a vertical area appears with a different color intensity than the reference area. However, positioning the samples with the convex side facing the IR camera resulted in higher thermal contrast—Figure 11. The temperature distribution along the measurement profile shows a clear temperature increase in the wrinkle area. The maximum thermal contrast was approximately 6.2 °C and 4.5 °C for the hand lay-up samples oriented to the convex and concave sides, respectively. Such high contrast makes thermography a convenient way to detect damage even with less accurate IR cameras (mobile systems, cell phone attachments, or cameras attached to drones).

In addition, the temperature distribution along the profile on the convex side, where the wrinkle is not visible to the observer, makes it possible to more accurately determine the area associated with the defect. When the element is viewed from the concave side, the wrinkle area is wider. This is due to the irregular surface created when the wrinkle is formed by hand lay-up. 

Figure 12 shows the temperature distribution during the analysis for two characteristic areas. Point P1 refers to the recorded temperature changes in the area associated with the wrinkle, while point P2 refers to the temperature changes in a reference area located away from the wrinkle. The location of the measurement points is shown in Figure 11. The temperature curves in Figure 11a show the changes recorded at measurement points placed on the sample with the convex side facing the IR camera, while Figure 12b shows the results when the sample is oriented with the concave side. 

The graphs show a significant temperature contrast between the wrinkled area and the intact area. The largest differences are observed at the end of the heating phase of the sample (30 s of the analysis), where the wrinkled area has a significantly higher temperature. Qualitatively, both sample positions, i.e., with the convex or concave side facing the IR camera, give similar characteristics. However, the setting with the concave side reduced the thermal contrast between the wrinkle and the reference area. This is due to irregularities in the wrinkle area that altered the heat flow through the sample. With the convex side (smooth side) setting, the higher temperature contrast is related to the significant increase in the sample thickness in the wrinkled area, rather than its irregularities as in the concave side setting.

**Figure 13:**
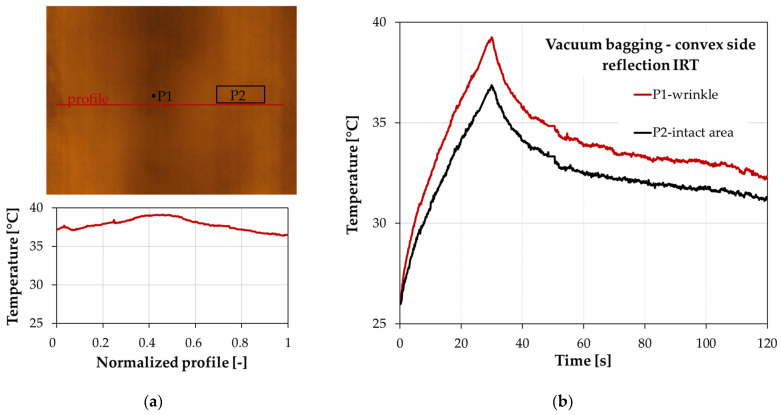
Thermal analysis from the convex side of the vacuum bagging sample: (**a**) thermal image and the temperature distribution along the profile at the end of the heating time and (**b**) the temperature distribution during the heating and cooling time for the wrinkle (point P1) and the reference area (point P2).

The thermographic results for the L-shaped sample produced by vacuum bagging are presented in Figure 13. As noted in Section 3.1, which focused on the quality of the sample—Figure 9, the artificially introduced wrinkle was significantly flattened as a result of the application of vacuum. In this case, the local thickness increase in the sample at the corner was about 0.3 mm. As a result, the thickness of the sample is not as severely disturbed in the area of the curve as it is for the hand lay-up sample. Because of the disappearance of the wrinkle, the thermographic analysis of the vacuum-bagged laminate was conducted to determine the influence of the curvature of the sample and the resulting difficulties in heating the sample uniformly, rather than to detect the wrinkle. Therefore, the vacuum-bagged sample can be treated as a wrinkle-free reference sample because of the wrinkle smoothing. With this assumption, the results obtained for the vacuum-bagged sample can be used to assess the effect of curvature on the thermographic results for the hand lay-up sample. This issue is discussed in more detail in Section 4.

Figure 13a shows the temperature distribution along the measurement profile at the end of the sample heating cycle (the 30 s of the analysis) for the vacuum-bagged sample. The maximum temperature contrast between the wrinkle-related area and the reference area was 2.8 °C. Figure 13b shows the temperature changes for the characteristic points associated with the wrinkle (point P1) and the intact area (point P2). The area associated with the wrinkle has a higher temperature with a maximum reached at the end of the heating cycle. In Figure 13, point P1 refers to the wrinkle; however, in a situation where the wrinkle is flattened under a vacuum, it would be correct to refer to this area as just the corner of the sample. However, to keep the descriptions clear, the name wrinkle was also used for the vacuum-bagged sample. 

The results for the sample made by the vacuum bagging technique are shown only for the convex side because similar temperature characteristics and temperature contrasts were obtained for the concave side. The similarity in the results is related to the similar smooth structure of the surface in the wrinkle area on the convex and concave sides. The situation was different for the hand lay-up sample, where the changes in the temperature contrast for the sample from the convex side compared with the results from the concave side amounted to almost 2 °C—Figure 12.

It should be added that in the case of small defects, there is a risk that the defect will not be detected. However, the testing technique itself, the ease of interpretation of the results, and the mobility of the device allow any doubts to be resolved by changing the configuration of the detection system, i.e., changing the distance of the IR camera from the test object increases the test area, and changing the mutual position of the IR camera and the heating system can change the direction of heat flow, which can facilitate the interpretation of thermograms. 

## 4. Discussion

Wrinkles in curved laminate structures occur mainly when elements of significant thickness are produced. The results of the thermographic analysis indicate that the ability to detect manufacturing defects in the form of wrinkles in curved laminates is mostly related to a local increase in sample thickness. The positioning of the elements in the thermographic setup, i.e., the position of the test object in relation to the IR camera and how the object is heated, is of great importance. The issue that should be raised here is the question of uniformity in sample heating. This is particularly important for curved samples.

Our previous studies [7] demonstrated the effectiveness of thermography in detecting wrinkles in flat glass laminate structures and curved thin-walled structures, such as wind turbine blades. The results of thermography studies for reflection and transmission techniques were the subject of much discussion in those studies.

The current thermographic study used a reflective version of active thermography, i.e., the heat source and the IR camera are on the same side of the object being analyzed. This configuration corresponds to the tests that are carried out on the real objects. The thermographic results showed that it is possible to detect defects in the form of wrinkles in curved laminates of significant thickness, but the value of the temperature contrast depends on the thickness of the wrinkle. The temperature contrasts obtained are largely due to a change in the thickness of the laminate in the area of the wrinkle and not to its morphology, i.e., the local presence of voids and a resin-rich area. The local difference in the morphology of the laminate is shown in the micrographs—Figure 9. 

Analysis of the effect of the position of the sample in relation to the IR camera showed that for curved laminates with significant wrinkle thickness, identification was more accurate when the sample was positioned on the convex side. The convex side is the smooth side of the sample that was in contact with the mold during manufacturing—Figure 2. This is important information because when detecting manufacturing defects on real objects, such as curved sections of a boat hull, the object is viewed from the side that was in contact with the mold.

Comparison of the thermographic results for a sample with a wrinkle and a sample without a wrinkle provides an opportunity to assess the effect of the method of heating the curved sample on the ability to detect damage and the value of the temperature contrasts. It can be assumed that the vacuum-bagged sample, in which the artificially introduced wrinkle was flattened, is the reference sample. With this assumption, it can be seen that the presence of a curvature in the L-shaped sample disturbs the uniformity of the temperature distribution. The temperature contrast between the curved area and the flat arms was approximately 2.8 °C for the vacuum-bagged sample—Figure 12. Even if the magnitude of the temperature contrast for the laminate sample takes into account the effect of the curvature of the sample and the difficulty of heating the component uniformly, the resulting contrast is still significantly higher (6.2 °C) than for the sample without the wrinkle. This demonstrates the effectiveness of wrinkle detection using reflection thermography on thick curved laminates.

The study also investigated the influence of the manufacturing process on the formation of wrinkles in woven multilayered glass laminates. If a wrinkle was created during lamination, the vacuum bagging technique resulted in a flattening of the previously introduced wrinkle—Figure 8. Microscopic observation of the vacuum-bagged sample showed that there was no significant disruption to the constituent layers of the laminate as the wrinkle disappeared. This behavior suggests that the choice of a suitable manufacturing process is critical in reducing fiber waviness and wrinkling in thick laminates.

## 5. Conclusions

There are many difficulties associated with the detection of wrinkles in curved structures made of multilayered laminates. In this study, active infrared thermography was used to detect the presence of wrinkles. During the manufacturing process, artificial wrinkles were created in L-shaped laminates. The research carried out in this study can be summarized as follows:

- The manufacturing process can affect the disappearance of wrinkles in curved laminate parts. In this study, this situation occurred when a vacuum bagging technique was used. The flattening of the wrinkle had no significant effect on the internal structure of the laminate.
- Active thermography can be effectively used to detect wrinkles in thick curved glass laminates when such deformations locally increase the thickness of the sample.
- Wrinkles of insignificant thickness that occur in curved laminates are difficult to detect by thermography because it is not possible to assess whether the temperature differences are due to the presence of the wrinkle or to a lack of uniform heating of the sample.

Further research will involve experimental analysis and mechanical modeling of curved glass laminates with wrinkles made from woven fabrics using out-of-autoclave techniques. Also, the possibility of formulating a qualitative relationship between the observed temperature and defect size in curved samples is planned in our future work.

## Data Availability

Data are contained within the article.
